# Editorial: Ethnopharmacology in Central and Eastern Europe in the Context of Global Research Developments

**DOI:** 10.3389/fphar.2019.00341

**Published:** 2019-04-09

**Authors:** Devesh Tewari, Judit Hohmann, Anna K. Kiss, Judith M. Rollinger, Atanas G. Atanasov

**Affiliations:** ^1^Department of Pharmacognosy, School of Pharmaceutical Sciences, Lovely Professional University, Phagwara, India; ^2^Department of Pharmacognosy, University of Szeged, Szeged, Hungary; ^3^Department of Pharmacognosy and Molecular Basis of Phytotherapy, Medical University of Warsaw, Warsaw, Poland; ^4^Department of Pharmacognosy, University of Vienna, Vienna, Austria; ^5^Institute of Genetics and Animal Breeding of the Polish Academy of Sciences, Magdalenka, Poland; ^6^GLOBE Program Association (GLOBE-PA), Grandville, MI, United States

**Keywords:** pharmacognosy, phytochemistiy, pharmacology, ethnobotany, ethnopharmacology

Historically, medicinal plants have been a key element of healthcare, and are still widely used as alternative and complementary therapy (mainly in developed countries) or as a primary treatment (in most developing countries). Moreover, many of the modern pharmaceuticals were developed from molecules extracted from natural sources, and medicinal plants still today represent an important pool for the identification of novel drug leads.

The ethnopharmacological tradition in Central and Eastern Europe has a great historical significance and large parts of the Western world's knowledge for therapeutic properties of medicinal plants has its roots in the Greek and Roman cultures (e.g., with a reference to the influential works of Dioscorides, Pliny the Elder, and Galen). The German-speaking Central European areas also have played very important roles, with some influential medieval herbal book editions such as the Mainz Herbal (Herbarius Moguntinus, 1484) and The German Herbal (1485). Moreover, at the beginning of the nineteenth century modern drug discovery from plants started to played a key role in Central Europe, most notably the work of the German apothecary assistant Friedrich Sertürner, who isolated an analgesic and sleep-inducing agent from opium that was named morphium (morphine) after the Greek god of dreams, Morpheus. At the same time, it should be noted that on many occasions the rich ethnopharmacological knowledge of some East European countries and Russia remained localized and did not find its way to integrate with the Western world of herbal therapy traditions. The focus of this Research Topic is to make a special contribution to ethnopharmacology rooted in Central and Eastern Europe, on the context of global research developments in the area of ethnopharmacology, phytochemistry, molecular pharmacology of natural products, nutrigenomics, and other related disciplines. This special issue consists of 24 articles covering diverse topics related to the listed research fields.

Advances in screening methods and analytical equipment, increasing number of targets available for testing, and improved possibilities for optimization of natural leads using synthetic modification strategies substantially improved the process of modern drug discovery from natural sources (Atanasov et al., 2015). *Hypericum* is a well-known genus that is used for its medicinal properties. Tocci et al. evaluated five *Hypericum* species for their polyphenolic content, toxicological safety, and antifungal potential against some sensitive and drug resistant clinical fungal isolates. The authors have identified 52 compounds by LC-MS analysis and showed that *H. hircinum* subsp. *majus* infusion exerted broad antifungal activity against *Aspergillus, Penicillium*, and non*-albicans Candida* strains (Tocci et al.).

Ethnobotany has played an important role in new drug development and drug discovery (Kayser, 2018). The importance of the traditional use of medicinal plants on the Greek islands of the North Aegean Region was summarized in a Brief Research Report Article by Axiotis et al. In this report the authors present 109 wild plants from 52 families, and enlist their therapeutic uses, including uses in galenic preparations which were reported by local medical doctors and pharmacists (Axiotis et al.).

Medicinal plants and natural products are of potential importance in several age-related disorders, including memory impairment and dementia (Tewari et al., 2018). In this research topic Dimpfel et al. assessed the biological activity of some commercial extracts of the underground parts of *Rhodiola rosea* L. and its analytical markers, salidroside and rosavin, using long-term potentiation of synaptic transmission in hippocampus slices (a synaptic model of memory).

Loeser et al. evaluated the effect of Casperome®, an orally bioavailable soy lecithin-based formulation of standardized frankincense extract on various systemic parameters and organ damages that were induced by severe systemic inflammation caused by intraperitoneal administration of lipopolysaccharides (a murine model of sepsis). The study revealed that the extract possesses anti-inflammatory, anti-oxidant, and hepatoprotective effects (Loeser et al.).

Zielińska et al. review older and recent data on pharmacology, phytochemistry, and clinical studies of *Chelidonium majus* L. along with controversies about this herb, its safety, and drug quality issues.

The study of Wozniak et al. evaluated the effects of phytochemically characterized extracts of various parts of traditionally used *Syringa vulgaris* L. (common lilac) on the pro-inflammatory functions of neutrophils. The authors also isolated active compounds, including neooleuropein from the extracts using bioassay-guided fractionation. It was found that neooleuropein acts through inhibition of cytokine production by attenuation of the MAP kinase pathways (Woźniak et al.).

Smooth muscle cells are a prominent target of diverse natural products with different bioactivities (Hnatyszyn et al., 2003; Uhrin et al., 2018). Sándor et al. assessed the relaxant effects or contracting activity of the hydroethanolic extract, fractions, phytoconstituents, and essential oil of Roman chamomile on smooth muscle preparations. The authors found that the essential oil of the plant possessed a good smooth muscle-relaxant effect (Sándor et al.).

Identification of the molecular mechanism of action of promising natural products is a vital step toward their clinical application (Butler, 2008; Tewari et al., 2018). In a mechanistic study, Celińska-Janowicz et al. studied the apoptosis-inducing mechanisms of selected propolis components in tongue squamous cell carcinoma cells (CAL-27). In another work, Grienke et al. isolated several flavonoids and rare sesquiterpene lactones from the ethanolic extract of *Centaurea ragusina* L., a plant species endemic to Croatia. For the first time they identified ragusinin, and hemistepsin A from this genus. The authors further studied the mechanism underlining the ragusinin activity in cancer cells (Grienke et al.).

In their review article Kozlowska et al. summarize the herbal medicines used in the Beskid mountain ranges, which are located south of Krakow and Lviv, two important medieval centers of apothecary tradition in the area. In another interesting review Kunnumakkara et al. summarize potential benefits, traditional uses, chemistry, biological activities, and clinical trials of “guggul” from *Commiphora* and *Boswellia*.

Berberine is an important phytoconstituent of plants utilized in different traditional medicine systems, including the Chinese system of traditional medicine (Huang et al., 2011). In their work “Berberine: Botanical Occurrence, Traditional Uses, Extraction Methods, and Relevance in Cardiovascular, Metabolic, Hepatic, and Renal Disorders” Neag et al. review diverse biomedical information on berberine, including several chromatographic techniques for berberine extraction and quantification.

There is great interest of the scientific community toward the health benefits of food items used in the Mediterranean diet, which has a variety of health benefits (Sofi et al., 2010). Wang et al. review recent studies on pomegranate, mainly focusing on vasculoprotective effects attributed to diverse pomegranate phytoconstituents, including tannins, especially ellagitannins, and ellagic acid, among other compounds. Lycopene is a carotenoid which is responsible for different pigmentation in tomatoes, grapefruit, and watermelon, and it represents a compound with a potent antioxidant action (Horvitz et al., 2004). In their work, Mozos et al. review mechanisms of action of lycopene on the vascular system considering substantial epidemiological and experimental data, and clinical studies.

Breast cancer is the most common cancer in women and represents a global health burden both in the developed and developing countries. Hong et al. study apigenin and luteolin to better understand their mechanism of action in the context of breast cancer intravasation of the lymphatic barrier. Both of these natural compounds were evaluated in a three-dimensional assay system containing MDA-MB231 breast cancer spheroids and immortalized lymph endothelial cell monolayers (Hong et al.)

Toiu et al. evaluated the chemical composition, antimicrobial, antioxidant, and *in vivo* anti-inflammatory activity of various extracts of the aerial parts of an important plant species with traditional use in Romanian, *Ajuga laxmannii* (Murray) Benth.

In their work, Shikov et al. review 70 wild plant species that are referenced in the 11th edition of the State Pharmacopeia of the USSR and also discuss their health food value, mainly based on published Russian literature.

Marchelak et al. present a comprehensive study focused on extracts of the flowers of *Prunus spinosa* L. The authors profiled the phenolic contents of the extracts through UHPLC-PDA-ESI-MS^3^, and also studied several biological effects with relevance in the context of cardiovascular disease (Marchelak et al.).

Chowdhury et al. explore the anti-proliferative and anti-metastatic potential of various extracts of the medicinal plant colocynth. The authors have found that ethanol and acetone extracts of colocynth fruit pulp exhibit promising activity in different cancer cell types, inhibiting viability, and cell migration in association with the modulation of the expression of diverse relevant genes and cellular pathways (Chowdhury et al.).

*Ononis natrix* is a member of the Fabaceae family and is a less-explored plant species. In their work, Yerlikaya et al. give new insight on the chemical composition and biological activities of *Ononis natrix* subsp. *hispanica*.

Wang et al. identify and characterize the natural product falcarindiol as a novel inducer of macrophage cholesterol efflux, a process with a protective effect in atherosclerosis.

In their research paper, Raclariu et al. establish DNA metabarcoding and HPLC-MS techniques for the authentication of *Veronica officinalis* L. herbal products. The authors advocate for the use of an integrative approach for the detection of adulteration and substitution in the herbal products (Raclariu et al.).

López et al. study the effects of lavender essential oil on pharmacological targets that are involved in anti-depressive and anxiolytic effects, as well as on *in vitro* models of neurotoxicity.

Lewenhofer et al. performed an activity-guided identification of active principles from *Scrophularia lucida* L. using cancer cell models. The authors identified 14 substances including some very rare iridoids, like scrovalentinoside or koelzioside, and flavonoids such as nepitrin and homoplantaginin (Lewenhofer et al..)

In conclusion, this Research Topic includes a wide range of interdisciplinary research work contributing to the ultimate goals of augmenting the existing knowledge and fostering the scientific field of ethnopharmacology in Central and Eastern Europe, as well as globally. This research topic successfully gathered comprehensive interdisciplinary information in the field of ethnopharmacology from the field surveys to standardization and elucidation of molecular mechanisms of action of natural products. Moreover, with consideration of the existing challenges in the field of drug discovery form medicinal plants, this research topic provides up-to-date snapshot of current knowledge and paves the way for further chemical and biological assessment of diverse natural compounds with potential for future therapeutic development. We hope that this compendium will further inspire scientists from different research fields to make use of the gathered traditional medical knowledge in the search for superior future remedies from nature.

## Author Contributions

All authors listed have made a substantial, direct and intellectual contribution to the work, and approved it for publication.

### Conflict of Interest Statement

The authors declare that the research was conducted in the absence of any commercial or financial relationships that could be construed as a potential conflict of interest.
